# Depression, anxiety, and drug usage history indicators among institutionalized juvenile offenders of Brasilia

**DOI:** 10.1186/s41155-021-00184-x

**Published:** 2021-06-22

**Authors:** Manuella C. da Silva, Antonio Pedro M. Cruz, Maria O. Teixeira

**Affiliations:** 1 Faculty of Psychology, University of Lisbon, 1649-013 Lisbon, Portugal; 2 Institute of Psychology, University of Brasilia, Brasília, Distrito Federal 70910-000 Brazil

**Keywords:** Juvenile offenders, Depression, Anxiety, Drug abuse, Mental health

## Abstract

**Introduction:**

One of the most serious socio-educational measures for children and adolescents in conflict with the law in Brazil is their internment. This measure may represent an additional source of stress to this population and present significant impacts in the mental health context. This study aims to describe anxiety levels, depression, and addictive consumption, as well as to estimate the causalities and interactions of these variables.

**Methods:**

Herein, we report a study in which 175 male juveniles from youth detention institutions of the Federal District voluntarily completed the Beck Depression Inventory (BDI), the Beck Anxiety Inventory (BAI), and a short self-rating questionnaire asking whether and how often they had experienced cannabis, alcohol, and other “hard” psychotropic drugs (e.g., crack, cocaine, amphetamine) 1 year prior to institutionalization.

**Results:**

Of the total participants, 28.00% showed moderate to severe depression scores and 34.28% showed moderate to severe anxiety scores. In addition, the vast majority of participants also reported some antecedent drug abuse, including cannabis, alcohol, and other “hard drugs.” The BDI scores moderately correlated with BAI, but none of these parameters considerably correlated with the antecedent drug abuse.

**Conclusions:**

The data indicate potentially concerning levels of emotional distress in these institutionalized juveniles which seem to be independent of addictive behaviors. These data deserve attention and further investigation. Thus, a need for preventative mental health for the general population and socio-educational intervention aimed at interned youth which can decrease levels of emotional stress is emphasized.

## Introduction

Brazilian Federal Law 8.069, known as the Statute for Children and Adolescents (ECA, 1990) and the National System of Social and Educational Assistance (SINASE, 2012) regulate juvenile justice in Brazil. Children and adolescents aged 12 to 17 years who commit an offense are held responsible through socio-educational (protective) measures, which may vary from the least serious to the most serious. Deprivation of liberty for up to 3 years in youth detention institutions with a compulsory release at the age of 21 years is the most serious of these socio-educational measures and should be applied in compliance with the principles of exceptionality and brevity.

Despite internment in youth detention facilities complying with principles of exceptionality and brevity, the number of children and adolescents subjected to this socio-educational measure in Brazil is very high. As recently pointed out in the Brazilian Annual Survey of the National Socio-Educational Service System (SINASE, 2018), Brazil had 26.868 young offenders distributed in 418 exclusively male socio-educational units. This incidence is not only due to the violence of the offense committed for the first time by these juveniles (e.g., homicide, robbery, armed violence), but also because many of them often remain in the youth detention institution until the maximum sentence date (3 years) or become repeat juvenile offenders.

The characterization of socio-educational measures in Brazil portrays the precariousness of the physical structure and activities offered, as well as a coercive organizational climate marked by hostile interpersonal relationships and insecurity, physical and psychological violence, as well as situations of conflict, escape, rebellion, mutiny, and homicides. There seems to be a prevalence of coercive practices over pedagogical ones. These places have been repeatedly described as overcrowded and unfavorable to development due to unsatisfactory and inhumane hygiene, food, health and physical integrity conditions, which results in the loss of personal identity, suffering, recurrence, and prejudice in the community. The comparison with prisons was frequent due to the prevalence of closed spaces, high walls, plurality of gates, twisted wires, and other security structures, as well as the difficulty of the adolescents to live with their family and community (Coscioni, Costa, Rosa, and Koller, 2017).

The history of slavery, genocide of native populations, and democratic fragility are important aspects for Brazilian political and social understanding (Kilduff, and Silva, Mossicléia Mendes da., 2019). The increase in incarceration and the physical extermination of black adolescents in Brazil (Gorski and Cordeiro, 2018) is the result of the “fear culture” reinforced by media appeals (Azevedo and Rebouças, 2017). In addition to the precariousness imposed by poverty and the lack of public policies, the social invisibility of young people and the appreciation of the culture of money, power, violence, and consumption linked to illicit drug trafficking influence the adhesion of young people to criminal practices. Complementing these ideas, Bonalume and Jacinto (2019) emphasize the selectivity of justice in the face of a society deeply marked by social, gender, race, and ethnic inequality.

International studies (e.g., Desai et al., 2006; Fazel, Doll, and Långström, 2008; Gretton and Clift, 2011; Grigorenko, Edwards, and Chapman, 2015; Livanou, Furtado, Winsper, Silvester, and Singh, 2019; Penner, Roesch, and Viljoen, 2011; Schubert, Mulvey, and Glasheen, 2011; Steiner, Garcia, and Mathews, 1997; Teplin, Abram, McClelland, Dulcan, and Mericle, 2002; Teplin, Welty, Abram, Dulcan, and Washburn, 2012) have identified a substantial proportion of psychological problems in institutionalized juvenile offenders. Depression, anxiety, and substance abuse are among the most frequently reported disorders. However, in many cases, it is unclear whether the juveniles already had these problems prior to institutionalization or whether the deprivation of liberty contributed by itself to developing or worsening their symptoms. In either case, the mental health needs of this population are unquestionable.

On the other hand, studies in the general population of young people and adolescents show that the beginning of psychoactive consumption is increasingly precocious and with heavier substances (Chiapetti and Serbena, 2007). These authors note that the most frequent uses in a higher education environment include alcohol, tobacco, marijuana, and stimulants. Family adversity and dysfunction and delinquent colleagues are factors leading to drug use. On the other hand, adolescents who feel inserted in the community and have positive expectations from educators are more protected. In the mental health context, risk factors for addictive behaviors are the insecurities which characterize adolescence, as well as the family, social and academic environment characteristics.

The literature (e.g., Abilleira, Rodicio-García, Vázquez, de Deus, and Cortizas, 2019; Teixeira and Costa, 2017) shows gender differences in emotional vulnerability, with women presenting higher anxiety levels, depression, and neuroticism. The community, the group of friends, and the family can function as protective factors in the relationship between emotional difficulties and consumption of psychoactive substances, but also as risk factors. According to Chiapetti and Serbena (2007), the interventions themselves are factors which contribute to a positive change in the relationship between mental health, drug use, and quality of life (Limberger and Andretta, 2018). In socio-educational institutions, the pedagogical assistance recommended by the law (Volpi, 2015) must contemplate the mental health of young offenders, as the institution itself represents an additional source of stress, which can further affect juvenile psychological distress.

Social risk factors seem to be prevalent, including in studies on psychopathy with Brazilian adolescents (Davoglio, Gauer, Vasconcellos, and Lühring, 2011; Castellana, de Barros, and Serafim, A. de P.,, and Busatto Filho, G., 2014). A study by Barros et al. (2013) carried out with adolescents interned in São Paulo indicated that no individual in their sample met the formal criteria for psychopathies based on the parameters of the *Psychopathy Checklist Revised Inventory*, reinforcing the prevalence of environmental aspects about inherited aspects. In another study involving adult prisoners, low social class and lack of education of the participants, as well as an unhealthy environment, lack of social assistance and health, and lack of credibility to the complaints of the interviewees are factors which influence the reported “depressive symptoms, followed by anxiety, and later psychosomatic symptoms” (Coelho, 2012).

In contrast to the great number of international studies evaluating depression, anxiety, and drug abuse profiles in institutionalized juvenile offenders, relatively few empirical studies have addressed these parameters in similar Brazilian samples (Areas-Arêas Neto, Constantino, and Assis, 2017), namely in Brasilia, the capital city of Brazil. Thus, the main aim of the current study was to identify the presence and intensity of depression and anxiety indicators and drug usage history in juvenile offenders in youth detention institutions in the city of Brasilia and its surroundings of the Federal District, Brazil.

## Methods

### Participants

Brasilia and its surroundings of the Federal District- DF, Brazil, contain a total of six youth detention facilities exclusively for male juveniles. At the time of the study, there were 579 institutionalized youth (Coordenação de Internação, Subsecretaria do Sistema Socioeducativo, Secretaria de Estado de Justiça e Cidadania do Distrito Federal, personal communication, October 26, 2020). This study included 175 male juveniles from three of these institutions who were voluntarily recruited by convenience and accessibility.

In order to characterize the sample presented, it is extremely important to consider that adolescents are interned as the last possibility of applicable socio-educational measure. This means that the other possible socio-educational measures (warning, obligation to repair the damage, provision of services to the community, assisted freedom, and semi-freedom) were applied and did not achieve the desirable effects characterizing the adolescents as repeat offenders or that there was a “committed infraction of serious threat or violence to the person” (Article 112, ECA, 1990). Thus, our entire sample of adolescent offenders is characterized by repeat offenders and/or those who have committed more serious crimes or violence.

Table 1 presents the final demographic composition of the studied sample. The mean (Mean ± SD) age of the participants was 16.80 ± 1.23 years (range 14–20), with a mean internment duration at the youth detention institution of 10.73 ± 7.36 months. The mean years of formal education were 8.44 ± 1.88, which is equivalent to elementary school in Brazil.

**Table 1:** Demographic characteristics of participants

Sample characteristics (all male juveniles)	
Participants	n = 175
Mean (± SD) age (years)	17.00 ± 1.00
Mean (± SD) years of schooling	8.44 ± 1.88
Mean (± SD) months of internment	11.00 ± 7.00

The protocol for this study was approved by the National Ethics Committee in Research, Brazil (Protocol CAEE 34895287), in compliance with the Code of Ethics of the World Medical Association (Declaration of Helsinki).

### Instruments and procedures

All participants who agreed to participate in the study were included in the sample and signed informed written consent prior to participation. Informed consent was signed by each participant in the case of adults, and the informed assent was signed by the heads of each Unit in the case of minors, in accordance with the recommendations of the Ethics Committee.

Legal authorization for data collection was obtained from the executive and judicial bodies of the Federal District. The interview schedules were pre-scheduled and agreed with by the managers (directors, safety, and psychosocial managers) so as not to interfere with the adolescents’ schedules and activities. The subjects were invited to participate in the study and no incentive was offered for participation.

The inclusion criteria for participation in the study were (1) being in a socio-educational measure with institutional internment; (2) availability to participate without prejudice to routine institutional activities; and (3) reading ability, considering that the instruments applied required a minimum level of reading and understanding. This selection was made by members of the Psychosocial Management of each Unit. Each group of adolescents was previously selected by the Security Management to not include young people with conflicts in the institution in the same room to avoid conflicts and fights during the application of the tests.

The researchers took strict care to preserve the confidentiality of the information and the confidentiality of the data, especially those related to the identification of the adolescents. Thus, this information was encoded for statistical handling and the original data was deleted.

Detailed information about the purposes of the study was provided for all participants. They were also explained that their personal information was anonymous, and their names were not used in the data collection instruments.

The participants completed the Brazilian validated versions (Cunha, 2001; Gorenstein and Andrade, 1996; Gorenstein and Andrade, 1998) of the Beck Depression Inventory (BDI; Beck, Steer, and Brown, 1996) and the Beck Anxiety Inventory (BAI; Beck, Epstein, Brown, and Steer, 1988; Beck and Steer, 1993) to measure the presence and intensity of depression and anxiety indicators. These two inventories are widely used in general population samples of both adults and juveniles, in addition to being used on patients who meet clinical diagnostic criteria for depression or anxiety (Borden, Peterson, and Jackson, 1991; Osman, Barrios, Aukes, Osman, and Markway, 1993; Steer, Ranieri, Beck, and Clark, 1993).

The BDI consists of a 21-item self-rating instrument in which subjects are asked to rate the presence and intensity of depression symptoms based on four response choices ranging from 0 to 3. The total score reflecting depression may vary from 0 to 63. The BAI is a 21-item self-rating inventory meant to measure anxiety. Each item is rated on four response choices (0 to 3) which reflects increasing severity levels of anxiety-related indicators. The total score reflecting anxiety may vary from 0 to 63. The BDI and BAI show high internal consistency, as indicated by Cronbach’s alpha coefficients of 0.81 and 0.92, respectively (Beck et al., 1988; Gorenstein and Andrade, 1998).

Next, the total BDI and BAI scores were calculated for each participant and subsequently rated into relative cut-off ranges used to categorize the depression and anxiety degrees according to the original guidelines for the BDI and the BAI instruments (Beck, Steer, and Garbin, 1998; Beck and Steer, 1993; Beck et al., 1996). The cut-off scores for the BDI were 0–9 (*minimal depression*), 10–16 (*mild depression)*, 17–29 (*moderate depression*), and 30–63 (*severe depression*). The cut-off scores for the BAI were 0–7 (*minimal anxiety*), 8–15 (*mild anxiety*), 16–25 (*moderate anxiety*), and 26–63 (*severe anxiety*).

The participants were then asked to fill in a short self-report questionnaire to assess their antecedent drug use profile, asking whether and how often they had experienced cannabis, alcohol, and other “hard drugs” (i.e., crack, cocaine) one year prior to institutionalization. Although somewhat vague, the term “hard drugs” was used herein to get closer to the colloquial language used by these juveniles to refer to these drugs, which mainly include crack and cocaine, but also methamphetamines and solvents. Only data from participants who reported recurring (≥ 3 times) use of any of these substances in their lifetime were included as drug users. Participants who reported never having used cannabis, alcohol, or other “hard drugs” and those who reported having used some of these substances up to three times during their lifetime were all counted as non-users.

Small groups of participants (2–17) were organized and sent on different days to a spacious room (usually a classroom within the institution) with the presence of the researcher (a psychologist with expertise in institutionalized juvenile offenders) and one member of the institution’s security staff to complete the instruments.

The percentages, average values, and standard deviations for each of the variables studied were calculated. The Pearson’s correlation coefficient for BDI, BAI, and drug use values was additionally calculated. Then, multiple linear regression was performed to test the interaction models between the variables studied. The data were analyzed statistically using the SPSS version 26.0 software program.

## Results

Table 2 depicts the total scores for BDI and BAI according to its relative cut-off ranges used to categorize individuals with minimal, mild, moderate, and severe depression or anxiety.

**Table 2:** Mean (± SD) total scores on the Beck inventories for depression (BDI) and anxiety (BAI) according to the relative cut-off ranges used to categorize individuals with minimal, mild, moderate, or severe depression or anxiety

BDI scores (cut-off ranges)	(n)	%	BAI scores (cut-off ranges)	(n)	%
0–9 (minimal depression)	88	50.29	0–7 (minimal anxiety)	70	40.00
10–16 (mild depression)	38	21.71	8–15 (mild anxiety)	45	25.71
17–29 (moderate depression)	37	21.14	16–25 (moderate anxiety)	31	17.71
30–63 (severe depression)	12	6.86	26–63 (severe anxiety)	29	16.57

Of the total participants, 88 (50.29%) had total BDI scores ranging from 0 to 9 (minimal depression), 38 (21.71%) had scores from 10 to 16 (mild depression), 37 (21.14%) had scores from 17 to 29 (moderate depression), and 12 (6.86%) had scores from 30 to 63 (severe depression). For the BAI, 71 (40.00%) participants had total scores from 0 to 7 (minimal anxiety), 45 (25.71%) had scores from 8 to 15 (mild anxiety), 31 (17.71%) had scores from 16 to 25 (moderate anxiety), and 29 (16.57%) had scores from 26 to 63 (severe anxiety). Taking the relative cut-off ranges for depression and anxiety, moderate to severe depression accounted for about 28% of participants, while moderate to severe anxiety accounted for about 34.28% of participants.

In terms of antecedent drug use, 156 (89.14%) participants admitted to using drugs several times during the period correspondent to 1 year prior to liberty deprivation. Table 3 illustrates the types of drugs used with the following user profile: 58 participants (33.14%) reported using cannabis, 49 participants (28%) reported consuming alcohol, and 48 (27.42%) reported using “hard drugs.” Some participants reported using more than one drug, with the following profile: 20 (11.42%) reported using cannabis and consuming alcohol, 16 (9.14%) reported using cannabis and “hard drugs,” 33 (18.86%) reported consuming alcohol and using “hard drugs,” and 31 (17.71%) reported using all three classes of drugs. Lastly, 19 (10.86%) participants reported not having used any drugs.

**Table 3:** Drug use profile one year prior to institutionalization

Drug(s) used	(n)	%
Cannabis	58	33.14
Alcohol	49	28.00
“Hard drugs”	48	27.42
Cannabis + alcohol	20	11.42
Cannabis + “hard drugs”	16	9.14
Alcohol + “hard drugs”	33	18.86
All three	31	17.71
None	19	10.86

A Pearson’s correlation matrix was then performed to investigate whether there was any correlation between BDI, BAI, and drug use, which is summarized in Table 4. Moderate positive correlations were observed between the total scores for BDI and BAI (r = 0.54), between alcohol and “hard drugs” (r = 0.45), between cannabis and “hard drugs” (r = 0.36), and between cannabis and alcohol (r = 0.31). Neither BDI nor BAI scores considerably correlated with the antecedent use of any of these drugs (rs ≤ 0.30). The correlations between the three types of consumption show a coefficient of 0.40.

**Table 4:** Pearson’s correlation coefficient for BDI, BAI, and antecedent drug use of cannabis, alcohol, and “hard drugs”

	Cannabis	Alcohol	“Hard drugs”	BAI	BDI
Cannabis	–				
Alcohol	0.31*	–			
“Hard drugs”	0.36*	0.45*	–		
BAI	0.04	0.24*	0.12	–	
BDI	0.09	0.20*	0.10	0.54*	–

Note. Only correlations ≥ 0.3 are highlighted. Additional explanations in the text.

*Statistically significant correlation coefficients at the level of *p* < .05

Multiple linear regression models were estimated for the dependent variables of anxiety and depression using the enter method. This procedure intended to clarify the casualty between variables which interfere in the psychological functioning of the offending adolescents and that should be considered in the socio-educational intervention plans. The independent variables included the addictive behaviors of alcohol, cannabis, and “hard drugs,” and also depression and anxiety. The assumptions of normal distribution, multicollinearity, homogeneity, and independence of errors were considered. Significant effects are considered from p < .05.

The model for anxiety (Table 5) was statistically significant, explaining 30% of the variance [*F*(4,106) = 12.22; *R*^2^_a_ = .30; *p=* 0.000]. The VIF value varies between 1.46 and 1.02, and the Durbin-Watson value is 1.73. Depression (β = .49, p = .000) and alcohol consumption (β = .22; p = .03) were variables which interfere with anxiety levels.

**Table 5:** Synthesis of the multiple regression model (enter) for anxiety.

Predictors	Non-standardized coefficients		*t*
	β	Error	
Depression	.63	.11	5.89
Alcohol consumption	1.78	.80	2.22

The model for depression (Table 6) was statistically significant, explaining 25% of the variance [(*F*(4, 104) = 9.71; *R*^2^_a_ = 0.25; *p* = 0.000)]. Next, two outliers with potential impact on the estimation of the regression line were identified through the results from the Mahalanobis distance, with the model being calculated without these extreme values. The VIF value varied between 1.53 and 1.10, and the Durbin-Watson value was 2.01. The anxiety variable explained depression (β = .54; p = .000), with this data being predictable by the association between the two variables.

**Table 6:** Synthesis of the multiple regression model (enter) for depression

Predictors	Non-standardized coefficients		*t*
	β	Error	
Anxiety	.37	.06	5.89

## Discussion

Psychological distress and consumption of psychoactive substances among institutionalized juvenile offenders is a worldwide phenomenon. Even though Brazil has an updated protective legislation for juvenile justice, the number of Brazilian children and adolescents in this situation is high, and little is known about the prevalence and severity of their psychological distress and mental health needs. This study had the focus to identify depression and anxiety indicators in male juvenile offenders at youth detention institutions in the city of Brasilia and its surroundings in the Federal District of Brazil.

According to a 2017 survey by the World Health Organization (WHO, 2017), individuals with depression accounted for 5.8% and individuals with anxiety 9.3% of the Brazilian population. Our findings show that participants with moderate to severe scores on the BDI (28.00%) and participants with moderate to severe scores on the BAI (34.28%) present about four times these rates, which indicates potentially concerning levels of depression and anxiety in these institutionalized juvenile offenders. Similar prevalence of depression and anxiety have also been reported in justice-involved youth in several countries (Odgers, Burnette, Chauhan, Moretti, and Reppucci, 2005; Steiner et al., 1997; Teplin et al., 2002; Teplin et al., 2012; Wakefield, Baronia, and Brennan, 2019).

In line with previously reported studies (Alali, 2016; Beck et al., 1988; Fydrich, Dowdall, and Chambless, 1992; Steer et al., 1993), our results indicated a positive moderate correlation (r = 0.54) between BDI and BAI scores. This behavioral profile may be attributed to the fact that depression and anxiety signals sometimes coexist in the same individual (Kalin, 2020; Mineka, Watson, and Clark, 1998), although exploratory factor analysis results in samples of psychiatric patients have found BDI and BAI scores to load in two different factors (Hewitt and Norton, 1993).

Youth drug abuse is well-documented in different countries and cultures, including Brazil (e.g., Abilleira et al., 2019; Banken, 2004; Carliner, Brown, Sarvet, and Hasin, 2017; Galduróz, Noto, Nappo, and Carlini, 2005; Lopes, Nobrega, Del Prette, and Scivoletto, 2013; Somani and Meghani, 2016), but its rate and degree of severity in institutionalized juvenile offenders is even higher (Grigorenko et al., 2015; Martin and Pillon, 2008). Our results for the youth detention institutions of Brasilia in the Federal District of Brazil corroborate these general findings.

In the linear multiple regression models, only alcohol consumption positively influenced anxiety levels. People with anxiety and mood disorders are more likely to abuse alcohol, anxiolytics, and other illicit drugs such as cannabis and cocaine than in the general population (Christie et al., 1988; Colder, Lee, Frndak, Read, and Wieczorek, 2019; Miguel et al., 2017; Paulus, Hogan, and Zvolensky, 2018; Schubert et al., 2011; Spradlin, Marcus, and Cuttler, 2020). Intriguingly, data of actual research showed a significant (p < 0.05) but very weak correlation (rs ≤ 0.30) between the drug abuse and the total scores for either the BDI or BAI. Therefore, at least in this studied sample, this implies that juvenile drug abuse was not a result of their anxiety or depression, and vice-versa. As recently shown by King et al. (2020), internalizing traits such as anxiety and depression were associated with more frequent alcohol use in adulthood, but not in adolescence. However, in a cohort study, Patton et al. (2002) showed that frequent cannabis use in adolescents increased the risk for later depression and anxiety, but depression in adolescents did not predict subsequent drug use. Part of the discrepancies may also be due to a wide range of instruments used to assess depression, anxiety, and substance use, which include semi-structured interviews, psychiatric tests, and several validated and non-validated inventories.

The results indicated the existence of moderate positive correlations between alcohol and cannabis (r = 0.31) and between alcohol and “hard drugs” (r = 0.45). The combination of alcohol and psychostimulants such as cocaine, crack and amphetamines, or alcohol with cannabis, is well known and has been observed in clinical and non-clinical samples in adults and adolescents (Flanner, Morgenstern, McKay, Wechsberg, and Litten, 2004; Gossop, Manning, and Ridge, 2006; Pennings, Leccese, and De Wolff, 2002; Scheffer, Pasa, and Almeida, 2010; Schneider Jr, Ottoni, de Carvalho, Elisabetsky, and Lara, 2015; Singh, 2019; Winward, Hanson, Tapert, and Brown, 2014). This usage pattern can promote effects that extend from synergy, when one substance enhances the effects of the other, to compensation, where the effects of one substance attenuate or counterbalance the effect of the other. Although in the present study we did not analyze these two forms of use in more depth, our results also point to the existence of combined use of substances in the studied sample of institutionalized adolescent offenders.

Finally, juvenile offenders have specific characteristics that can contribute to drug use in a different way from the emotional factors that contribute to drug use in the general population. For example, most juvenile offenders who are sentenced for serious offenses (e.g., homicide, robbery, armed violence, drug trafficking) for the first time lived in communities with high rates of psychosocial stress, including a lack of social assistance, domestic violence, and a history of childhood adversity and emotional abandonment (Pyle, Flower, Williams, and Fall, 2020; Uceda-Maza and Alonso, 2017). Acute stress is known to lead to anxiety, but uncontrollable chronic stress precipitates the onset of depression disorders (Maier and Seligman, 2016; Nestler et al., 2002). Therefore, when these juveniles are faced with another source of environment stress such as liberty deprivation for long periods, their emotional distress may be severely aggravated, mainly considering that children and adolescents are still developing their coping mechanisms (Compas et al., 2014).

Another important issue is the fact that the current study included only male participants. In fact, the population of youth detention institutions worldwide, including Brazil, is overrepresented by juvenile males (SINASE, 2018). Considering that women are nearly twice as likely as men to experience depression and anxiety-related symptoms (Kuehner, 2017; Remes, Brayne, van der Linde, and Lafortune, 2016), our findings should not be generalized for all genders. In a recent meta-analysis study, Livanou et al. (2019) showed a highly significant prevalence of affective disorders in institutionalized females compared to their male peers. Therefore, further Brazilian studies identifying and comparing indicators for depression, anxiety, and substance abuse in institutionalized female and male juveniles may offer additional insights into the problem. Some of the Brazilian works on the subject confirm this prevalence in female juvenile offenders. For example, in a sample of 30 juveniles from a detention institution exclusively for female juvenile offenders in the city of Rio de Janeiro, Brazil, more than 50% of the participants were found to meet diagnostic criteria for depression, and about 70% for anxiety and drug abuse disorders (Andrade, Assumpção Júnior, Teixeira, and Fonseca, V. A. da S., 2011). In another similar empirical study with juvenile offenders on parole in the city of Rio de Janeiro, females were found to display more depression and anxiety indicators compared to their male peers (Andrade, Silva, and Assumpção Júnior, 2004). However, there was no difference between genders in the drug abuse profile in this same study.

As pointed by Costa and Silva (2017), the public policies in Brazil are disintegrated and characterized by a very high degree of medicalization, deficiency in transportation to the health network, psychologists focused on judicial and non-therapeutic issues, low supply of psychotherapy, and other therapies. The mental health treatment in the inpatient units seems to follow what Arêas Neto et al. (2017) called biomedical logic. The legal guidelines for healthcare for this population are very distant from the daily practice of monitoring these adolescents (Perminio et al., 2018). Interventions aimed at this group need to contemplate both the treatment in relation to drug use and the behavior of an infraction, encompassing all of their requirements (Limberger and Andretta, 2018).

Some limitations should be considered when discussing the results of this study. First, there was no access to medical records during the sample selection which indicated any previously existing diagnoses of psychopathologies. Therefore, it may be that possible participants with other pathologies may have been included among the depression and anxiety scores found. Second, although the sample of 175 participants approached approximately 1/3 of the total number of young male offenders fulfilling socio-educational measures for internment in Brasilia, our results should not be generalized to the universe of young offenders undergoing similar socio-educational measures in Brazil. Third, the presence of at least one member of the security team during the application of the inventories may have inhibited the adolescents in their free expression of ideas and feelings. Fourth, we cannot ignore the fact that because it is a cross-sectional study, we have time frame limits which prevent generalizing the data and reduce understanding the evolution of adolescent development. Finally, there are limitations arising from self-report questionnaires, as well as the influences of expectations of social desirability which can influence response biases characteristic of this type of sample.

## Conclusions

In conclusion, although the present study was not focused on clinical diagnosis or psychiatric and psychological interventions, our findings that institutionalized juvenile offenders exhibited potentially concerning levels of depression, anxiety, and prior drug abuse may be useful for targeting intervention measures to treat or even prevent the emotional distress in this population and other similar ones. Some previously reported Brazilian studies have indicated a prevalence of psychiatric medicalization instead of other types of therapy in juvenile offenders deprived of liberty (Nascimento, Uziel, and Hernández, 2018; Vilarins, 2014). The fact that depression and anxiety indicators showed to be independent of the drug abuse profile may contribute to better targeting of these actions.

Considering the independence and interactions of the variables presented, socio-educational projects can be benefited and improved. Socio-educational programs which integrate this reality into the body of their interventions, with specific actions for treating toxicological dependence and withdrawal symptoms, as well as in the use of information and harm reduction campaigns, will be beneficial in promoting health for these adolescents. In addition, socio-educational activities aimed at promoting physical activities, leisure activities, psychotherapy, or occupational therapies aimed at reducing symptoms of anxiety and depression and promoting well-being should receive due attention from the managing bodies of the national socio-educational system.

## Data Availability

The data that support the findings of this study are available on request from the corresponding author [MCS]. The data are not publicly available because they contain information that could compromise research participant privacy/consent.
