# Correction to: Depression, anxiety, and drug usage history indicators among institutionalized juvenile offenders of Brasilia

**DOI:** 10.1186/s41155-021-00189-6

**Published:** 2021-07-26

**Authors:** Manuella C. da Silva, Antonio Pedro M. Cruz, Maria O. Teixeira

**Affiliations:** 1 Faculty of Psychology, University of Lisbon, 1649-013 Lisbon, Portugal; 2 Institute of Psychology, University of Brasilia, Brasília, Distrito Federal 70910-000 Brazil

**Correction to: Psicol Refl Crít 34, 17 (2021)**

**https://doi.org/10.1186/s41155-021-00184-x**

Following publication of the original article (da Silva et al. 2021), a sentence “Correspondence concerning this article should be addressed to Manuella Costa da Silva, Faculty of Psychology, University of Lisbon, Alameda da Universidade, 1649-013, Lisbon, Portugal” is mistakenly added in the Keywords section due to a typesetting error. This should be removed.

The updated Keywords section is given below, and the original article (da Silva et al. 2021) has been corrected.

**Keywords**

Juvenile offenders, Depression, Anxiety, Drug abuse, Mental health
